# Continuous psychophysics for two-variable experiments; A new “Bayesian participant” approach

**DOI:** 10.1177/20416695231214440

**Published:** 2023-12-21

**Authors:** Michael Falconbridge, Robert L. Stamps, Mark Edwards, David R. Badcock

**Affiliations:** School of Psychology, 2720 University of Western Australia, Crawley, WA, Australia; 8664 University of Manitoba, Winnipeg, Canada; Research School of Psychology, 2219 Australian National University, Canberra, ACT, Australia; School of Psychology, 2720 University of Western Australia, Crawley, WA, Australia

**Keywords:** Bayesian inference, data analysis, continuous psychophysics, induced motion, adaptation

## Abstract

Interest in continuous psychophysical approaches as a means of collecting data quickly under natural conditions is growing. Such approaches require stimuli to be changed randomly on a continuous basis so that participants can not guess future stimulus states. Participants are generally tasked with responding continuously using a continuum of response options. These features introduce variability in the data that is not present in traditional trial-based experiments. Given the unique weaknesses and strengths of continuous psychophysical approaches, we propose that they are well suited to quickly mapping out relationships between above-threshold stimulus variables such as the perceived direction of a moving target as a function of the direction of the background against which the target is moving. We show that modelling the participant in such a two-variable experiment using a novel “Bayesian Participant” model facilitates the conversion of the noisy continuous data into a less-noisy form that resembles data from an equivalent trial-based experiment. We also show that adaptation can result from longer-than-usual stimulus exposure times during continuous experiments, even to features that the participant is not aware of. Methods for mitigating the effects of adaptation are discussed.

## Introduction

Trial-based methods have been a mainstay in psychophysics since its inception over 160 years ago (Prins et al., 2016; Wichmann et al., 2018). Recently, however, there has been a renewed interest in continuous psychophysical approaches that call for the sustained, active participation of the person taking part in the experiment (Ambrosi et al., 2022; Bonnen et al., 2015; Burge & Cormack, 2020; Grillini et al., 2021; Huk et al., 2018; Kuo et al., 2017; Lakshminarasimhan et al., 2020; Straub & Rothkopf, 2022).

This renewed interest is a result of various factors: the capacity for rapid response sampling by fast computers; the ability to adjust stimuli in real-time based on participant responses; the natural extension of research interests from highly controlled lab scenarios to naturalistic environments and behaviors; and advances in Virtual Reality and other immersive systems enabling realistic simulation of more natural environments. If proven reliable, continuous psychophysics approaches would offer several advantages. They would allow for the study of human perception and behavior under more natural conditions, would provide more engaging experimental experiences which would reduce effects associated with participant fatigue and distraction, and the data could be collected in a fraction of the time needed for trial-based methods (Huk et al., 2018).

An issue that needs to be addressed if continuous methods are to play a significant role in the field of psychophysics is data reliability. There are at least two extra sources of variability inherent to a continuous approach where stimuli are continually varied randomly and participants respond concurrently.

- There is high uncertainty associated with the stimulus as it must be varied continuously and randomly in order to avoid guessing of future stimulus states.
- There is greater variability associated with the action system. Rather than the constrained number of response options intrinsic to trial-based experiments (often binary) and a generous amount of time to respond, participants are forced to respond quickly in a continuous experiment and there is a continuum of response options.

Is there a way to utilize the potential benefits of a continuous approach while addressing reliability concerns? Data reliability was bolstered in the case of trial-based approaches with the advent of signal detection theory (SDT, Green & Swets, 1966) because it provides a reliable means of accounting for, and making use of, the variability that is inherent in trial-based data. This is accomplished by explaining the source of that variability and how it relates to key internal states in the observer (Swets et al., 2001; Szalma and Hancock, 2015).

A continuous equivalent of SDT is needed that accounts for the variability in continuous experiments (see Straub & Rothkopf, 2022: for a discussion of the inability of SDT to model participants in continuous experiments). In the present paper, we discuss a novel model of a participant in a continuous experiment that accounts for the noise in the participant’s responses. We show how it can be used to produce data that is as low in unexplained variability as that obtained from a trial-based experiment.

Before presenting the model, it is important to define a realm in which continuous psychophysics could play a useful role, as that will determine the general shape of the appropriate model. Just like the earliest proposed continuous psychophysics method called the Method of Adjustment (Fechner, 1860), modern continuous methods are unlikely to be well-suited for determining precise detection thresholds and discrimination thresholds (or “just noticeable differences”) because of the variability inherent in these approaches (Blackwell, 1952; Farell & Pelli, 1999). Accordingly, recent continuous approaches have been employed successfully for studies that don’t involve determining thresholds. Examples include the estimation of sensory noise while a participant continuously tracks the position of a well-above-threshold Gaussian blob moving on a plane (Bonnen et al., 2015; Straub & Rothkopf, 2022), the measurement of internal noise while a participant tracks an object moving through three-dimensional (3D) space (Bonnen et al., 2017), the perceived motion of an object in 3D space as a function of the delay between left and right eye images (Burge & Cormack, 2020), the approximation of internal sensory and action noise while a participant corrects for random changes in the numerosity and area of a cloud of dots (Ambrosi et al., 2022) and tracing the temporal evolution of a contrast-induced speed bias (Patricio Décima et al., 2022).

A currently unexplored application for continuous psychophysics is the fast exploration of relationships between two above-threshold stimulus variables. In principle, a continuous method could be applied to quickly map the relationship for any study of the effect of one above-threshold variable on another in a psychophysical study. Examples include the effect of orientation of a high contrast surrounding grating on the perceived contrast of a central grating, the perceived tilt of a vertical bar as a function of the proximity of a pair of surrounding tilted bars, the effect of the position of a visual stimulus on the perceived location of an auditory stimulus, and perceived driving speed as a function of proximity to guard rails.

In this paper, we examine how the direction of a moving background affects the perceived direction of a moving target. It has long been known that when a target (moving or not) is placed against a background that moves, the target will perceptually inherit a motion vector opposite to that of the background (Duncker, 1929; Farrell-Whelan et al., 2012; Reinhardt-Rutland, 1988). The effect is referred to as Induced Motion. For example, when a red target circle moves vertically upwards on a computer screen against a background blue frame moving to the right, the target will appear to move upwards, as expected, but also to the left, in a direction opposite to the motion of the background.

In our example experiment, we continuously varied the direction of the background in a random manner so that participants could not guess what direction it would move in next, and asked participants to adjust the target so that it always appeared to move vertically. The difference between the actual target direction and its nominal perceived direction (vertical) was taken as the size of the induced motion effect for a given background direction (Falconbridge et al., 2022; Farrell-Whelan et al., 2012; Zivotofsky, 2004). Participants in the experiment thereby engaged in what may be termed a continuous-correction task. This is similar to a traditional above-threshold nulling task, but where an aspect of the stimulus that affects perception is continuously and randomly perturbed.

We now briefly introduce a Bayes-optimal model of an experimental participant which we call the Bayesian Participant (BP) model. The BP is assumed to have an internal generative model of the processes occurring in the outside world that produce the continuous stimuli experienced. The BP updates estimates of world states using two sources of information: Its own predictions about how the world will change, and new sensory data. The weight given to each is determined by the estimated variance within each information channel. The weights are Bayes-optimal.

The estimates of world states so produced are compared with states representing the participant’s goal and any discrepancies between the two drive participant action. In the case of our example experiment, the world variable of interest was the direction of motion of a target. The internal estimate of that direction corresponds with the perceived direction experienced by a participant. If this direction is left or right of vertical then an ideal correcting action is planned. Planned actions are those that are expected to produce perceptions that are more aligned with the goal based on the BP’s model of its own action system and how its actions change the world. The difference between the *planned* actions and the *actual* actions of a participant is attributed to additive noise in the action system.

This is one sense in which our BP model differs from a Kalman filter. Although they are similar in that both estimate hidden world states using a Bayesian inference framework, the BP model goes further by estimating appropriate actions for meeting a goal. We will see that another sense in which they differ is that the BP model assumes constant internal and external noise variance during experimental sessions.

**Figure 1.**
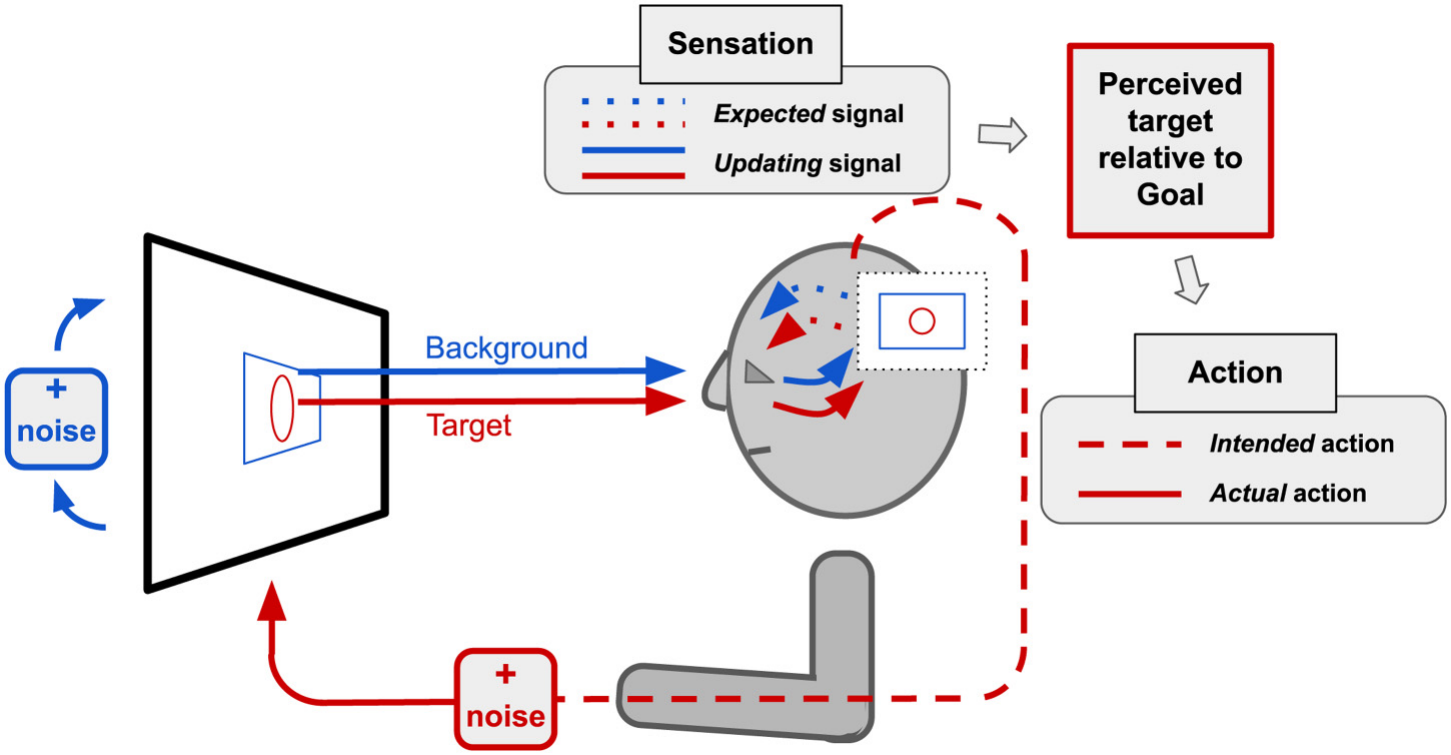
Schematic representation of our Bayesian Participant taking part in a continuous-correction experiment. The background (blue) direction is randomly perturbed (“+ noise”) at regular intervals and the target (red) direction is continuously adjusted by the participant in an effort to keep the perceived target direction close to a goal state. Estimates of world states (“Sensation”) and choices about how to correct the target (“Action”) are Bayes-optimal.

A schematic representation of the BP in the above context is sketched in Figure 1. We outline the BP model in more detail in the next section and provide mathematical details in Appendix A.

Our model has been designed to aid in the analysis of data from continuous experiments. The way it does so will now be described.

The first assumption for our data analysis approach is that the aim of a two-variable experiment to which our approach is applied is to probe the relationship between the dependent and independent variables. Recall that the dependent variable is the aspect of the stimulus that is altered by the computer, and the dependent variable is the aspect of the stimulus that is under the control of the participant. Updates to the second are made in response to the computer-driven adjustments to the first.

Such an experiment will produce data sets representing the dependent and independent variables, but the data will be corrupted by the extra sources of data variability outlined above.

Our second assumption is that there exists a mechanism in the brain of the participant that is the basis for the relationship between the dependent and independent data values. This mechanism can be thought of as a function; it takes current dependent and independent variable values and produces a “percept” for the dependent variable. In turn, this percept drives actions that determine future dependent values. We label this function f . We assume that this function f operates independently of the experimental approach; it will produce the same results whether the participant is in a trial-based experiment or a continuous experiment.

The differences between trial-based and continuous results lie in the sensory processes leading up to f and the action processes that follow f , not f itself. In a continuous experiment, the sensory system will suffer from the extra sources of perceptual variability outlined above and the action system will suffer from the extra sources of action variability outlined above. If these extra sources of variability can be accounted for, it should be possible to see clearly the operation of the function f by knowing its inputs and the percepts it produces at any one time. These function inputs and outputs should be the same as those in an equivalent trial-based experiment.

Our BP model makes precise predictions about how the dependent and indpendent variables will be altered by a participant’s sensory system during a continuous experiment to produce inputs to f . It also makes precise predictions about how the dependent variable percepts, which are the outputs of f , will lead to actions. This allows an experimenter to take the *actual* dependent and independent variable data sets and trace them downstream to find model estimates of inputs to the function f . It also allows an experimenter to trace the dependent variable data upstream through the action system to the model outputs of the function f that drove the actions of the participant. In this way, an experimenter can produce new data sets representing the dependent and independent inputs to f as well as the dependent outputs.

These new data sets are ideal because they are the values produced by a Bayes-optimal perception-action system. Our BP is similar to an ideal observer (Geisler, 2003), but with the addition of an “ideal” action system; one where the actions are optimal in a Bayesian sense. What we show below is that the ideal data sets produced using our BP model are similar to data sets from a more controlled trial-based experiment.

In the next section, we describe our BP model in enough detail that readers should be able to follow descriptions of its application in later sections. In the ‘Application of the BP Model to Induced Motion' section we detail our induced motion experiment and the application of the BP model to analyze the data from that experiment. In the ‘Comparing Results to Trial-Based Results' section we compare our results to those from an equivalent trial-based experiment conducted previously in our lab. The comparison is promising but it highlights a potential danger associated with continuous psychophysics approaches in general—the issue of adaptation. In our first experiment, participants were exposed to target motion for a prolonged period in one prevailing nonvertical direction. Even though the participants were unaware of this nonvertical component it appeared to cause adaptation in lower-level neurons which led to the participants adjusting the target more and more towards the prevailing nonvertical direction. Methods for controlling the impact of adaption are presented and shown to be effective. The paper closes with a short summary and discussion in the final section.

## BP Model

**Figure 2.**
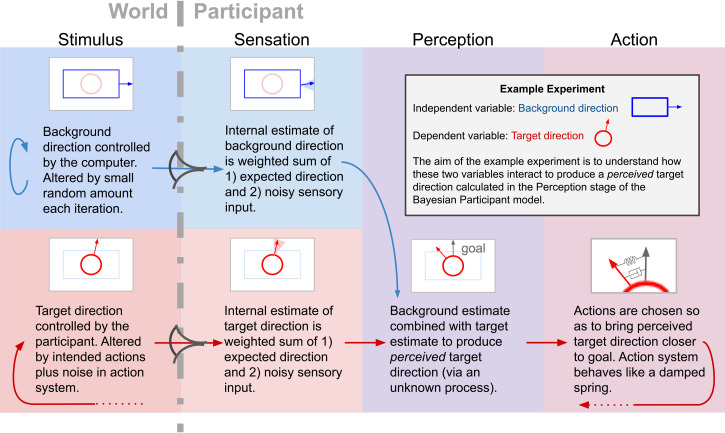
BP model in words. The background (blue) and target (red) update procedures are broken down into stages; changes to stimulus properties (World), the transduction of those properties into sensory states, the estimation of world stimulus properties based on sensations and an internal model of the external world (Sensation), the combining of background and target estimates to produce target perceptions (Perception) and the instigation of actions that drive target perceptions closer to goal states (Action).

**Figure 3.**
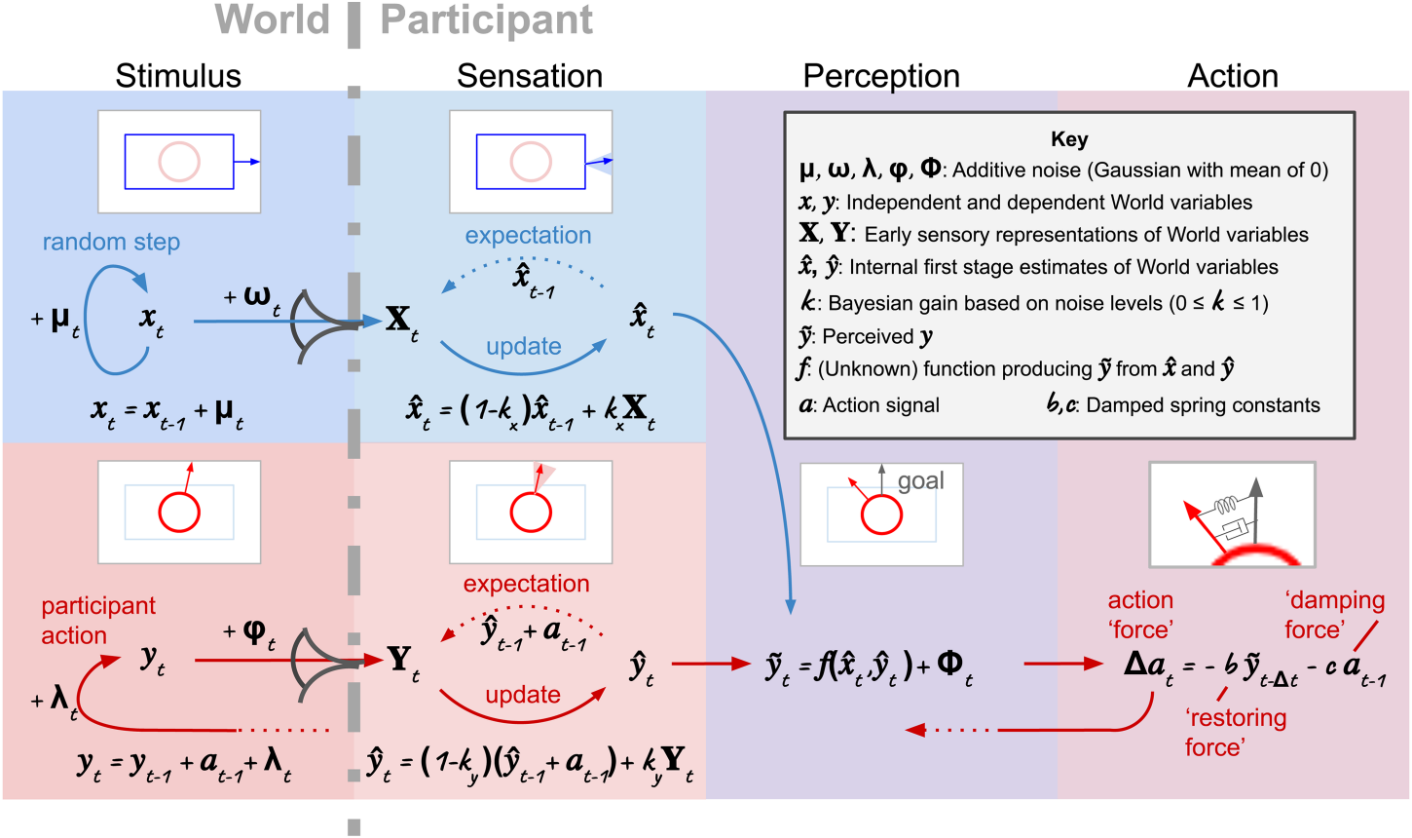
BP model in equations. Here, the words in Figure 2 have been replaced with equations. These equations describe all changes that occur at each iteration, both in the world and in the BP. See the text for an explanation and Appendix A for a deeper mathematical discussion along with the derivation of these equations.

The schematics in Figures 2 and 3 depict both a continuously varying stimulus and a BP responding to the changing stimulus in greater detail than does Figure 1. Figure 2 uses words to describe the stages, employing our experiment as an example, and Figure 3 contains the same stages represented in equation form. Arrows indicate loops and flows driving the stimulus and participant behavior.

As shown in the figures, the BP may be broken down into three stages. We have labeled these stages Sensation, Perception, and Action but note that these are lose categorizations; used only for the purpose of stage separation. Each of these stages will be treated individually below after the stimulus generative process has been described.

### Stimulus

The stimulus is composed of (at least) two parts one is controlled by the computer and the other is controlled by the participant. The variables representing the states of these components are labeled the “independent” and the “dependent” variables respectively. The word “dependent” is used because the perception of this variable is a function of the “independent” variable. The independent variable is labeled x in Figure 3 and the dependent is labeled y .

The independent variable is randomly perturbed at each iteration; stepped by a small amount, μ , where μ is selected from a Gaussian distribution with a mean of zero and variance $\sigma_{\mu }^{2}$ . The dependent variable is updated by the actions of the participant. These actions are the sum of an intended component, a , and zero-mean Gaussian noise, λ .

### Sensation

In the first stage of the BP model, denoted “Sensation” in Figures 2 and 3, estimates of x and y are inferred. These are labeled $\hat{x}$ and $\hat{y}$ respectively. Inference is based on Baye’s theorem. This stage employs a model of the dynamic behavior of the external world. This model makes predictions about future world states and has “expectations” about the future sensations that will flow from those predicted states.

The sensory expectations of the perceptual system are compared to the sensory data arriving from the world. The sensory data are labeled X and Y and are just noisy versions of x and y . If the prediction and sensory data differ, the internal estimate of the relevant external variable is updated. For the independent variable the statistically accurate expectation is that the sensory input will not change as changes to x in the world are random with a mean of zero. For the dependent variable, the statistically accurate expectation is that the previous state will be updated by the known actions of the participant.

The actual and expected sensory states are weighted by a weighting factor, k , and 1−k respectively. This factor is a simple function of the known variance in the sensory system and the estimated variance in the world variable. Calculation of k is detailed in Appendix A.

### Perception

The estimates of world states are combined in the Perception stage of the model where a “percept” of the dependent variable is formed based on an interaction between the estimates of the dependent and independent variables. This interaction between the independent and dependent variables is not known and the experiment is designed to probe it. For example, in our experiment, we examine target direction perception as a function of the current background and target directions. The internal estimate of target direction is labeled $\hat{y}$ , perceived target direction (nominally vertical) is labeled $\tilde{y}$ and the internal estimate of background direction is labeled $\hat{x}$ .

The interaction is described by the unknown function labeled $f(\hat{x},\hat{y})$ in Figure 3 which is a specific example of the function f used more generally in this paper. The inputs to this function are the estimates $\hat{x}$ and $\hat{y}$ from the previous stage of the BP model. The output is $\tilde{y}$ and represents the “perceived” value of the dependent variable.

The entire data analysis is centered on this stage of the model. The aim is to determine $\hat{x}$ and $\hat{y}$ from the raw data x and y and to determine $\tilde{y}$ from y based on a model of the actions of the participant. Calculating the inputs and outputs of $f(\hat{x},\hat{y})$ in this way allows us to determine the properties of this function experimentally.

### Action

The action system is based on the commonly used damped spring model of actions by human hands (André et al., 2014; Borst & Indugula, 2006; de Lussanet et al., 2002; Hollerbach, 1981; Speich et al., 2005). The function of the action system is to correct deviations of the perceived dependent variable from some goal state through the use of two forces. The first is a spring restoring force that “pulls” the perceived dependent variable towards the goal and the second is a damping force that slows movements to minimize overshooting.

The action system has two parameters: A spring constant b and a damping constant c . Note that a time lag Δt is included in the damped spring expression to account for the fact that responses to stimuli occur sometime after the driving stimulus is presented. The actions produced by the damped spring system are termed “intended actions” to distinguish them from the actual actions of the participant which have Gaussian noise, denoted λ , added to them. We attribute the noise to unexplained variability introduced by the physical system that implements the actions.

The actions are assumed to be driven by discrepancies between perceptions and goal states for the dependent variable. This shapes the experimental protocol. In our experiment, rather than have participants simply report the direction of the target stimulus under changing background conditions, they were instructed to adjust the target direction to meet a goal.

We propose that most phenomena involving relationships between two different stimulus properties can be studied using experiments where participants are tasked with adjusting some aspect of the stimulus to meet a goal. But, some phenomena may be more amenable to a tracking task where participants simply report the perceived state of some aspect of the stimulus. Reporting can take many forms, for example, pointing or moving a cursor on a screen to indicate the perceived position of some aspect of the stimulus. In Appendix D it is shown that our BP model can be adjusted slightly to be used in such experiments. This allows for target-tracking experiments like those used in most continuous psychophysics experiments to date (Ambrosi et al., 2022; Bonnen et al., 2015; Burge & Cormack, 2020; Kuo et al., 2017; Grillini et al., 2021; Huk et al., 2018; Lakshminarasimhan et al., 2020; Straub & Rothkopf, 2022).

Note that we have presented the action system independent of any Bayesian considerations. In Appendix C we show how the action system relates to Bayesian Decision Theory (BDT). Briefly, BDT assumes actions are chosen whose outcomes maximize utility. Utility is a measure of the worth of outcomes and takes energetic costs and the value of the outcomes into account (Körding & Wolpert, 2006). Using our experimental data we show that utility is approximately maximized by assuming the actions are a product of a damped-spring action system.

## Application of the BP Model to Induced Motion

**Figure 4.**
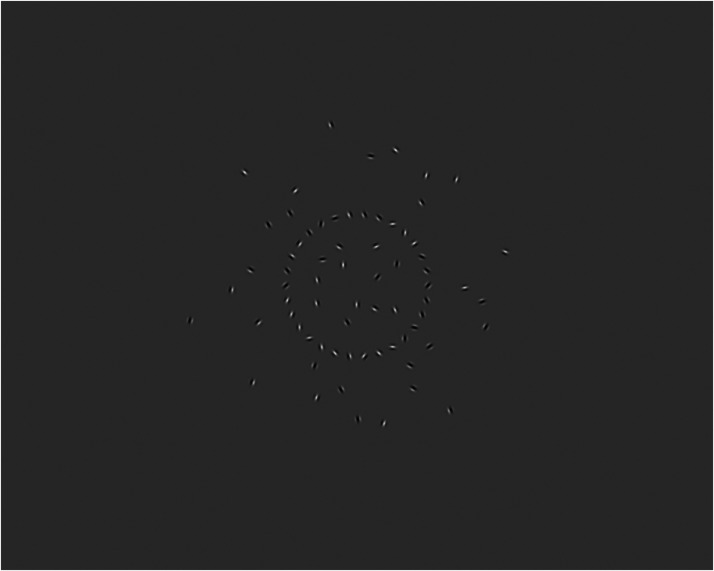
Screenshot of an example stimulus. The target consisted of 30 so-called “Gabor patches” arranged on a ring. Each stripy pattern within each stationary patch drifted in a manner consistent with the global target speed and direction. The remaining patches constituted the background. The patterns in these patches, likewise, drifted in a manner consistent with the global motion of the background. See example stimulus movie in Supplemental Materials 1.

In this section, we show how the Bayesian Participant model can be applied to a continuous version of an induced motion experiment. The independent variable, x , is the background direction and the dependent variable, y , is the target direction set by participants in an effort to make the target direction appear vertical. In our first experiment, this direction was generally up and to the right in order to cancel the leftward motion induced by the background that always has a rightward component of motion. A stimulus screenshot is shown in Figure 4. A movie of the stimulus is in Supplemental Materials 1.

The target consisted of 30 Gabor patches arranged in a circle centered on the middle of the display. Each Gabor remained in place but the pattern within drifted perpendicular to the orientation of its stripes at a speed that was consistent with the global velocity of the target object. The background consisted of the remaining 40 Gabors and the corresponding drift of each was consistent with the global background velocity.

Participants viewed this stimulus on a monitor and were asked to fixate as well as possible on the center of the display area while they continuously adjusted the target direction with a mouse to make it appear to move upwards. Fixating centrally is the best way to judge target direction as the nature of the stimulus calls for the integration of many differently oriented local patterns to get a sense of the global speed and direction of the target circle. This is because it is only possible to see a single component of the target motion by looking at a single Gabor patch, namely the component orthogonal to the Gabor’s stripes. This is because motion along the stripes is not visible as there is no texture to indicate such motion. One needs to integrate across at least two different component directions to get a sense of the 2D global target motion; the more such signals there are, the less noisy the global percept becomes. Global motion produced in this way is referred to as Global Gabor (Amano et al., 2009) or Dynamic Global Gabor motion (Rider et al., 2009).

The background elements also carried a global motion signal. The background direction underwent a random walk which affected the perceived target direction necessitating continuous corrections by the participant in order to keep the target perceptually moving vertically. Stimulus details are given in Appendix E.

The experiment described here is a continuous version of a trial-based experiment conducted previously in our lab (Falconbridge et al., 2022). The reasons for using a Global Gabor stimulus are presented in the original study. The data from the trial-based experiment, presented below as a comparison to our continuous experiment, took about 3.5 hours per individual to collect and produced 20 data points per individual. By way of comparison, the continuous method produced about 9 equivalent data points, based on about 2000 measurements, for each two-minute session. With this method, the same 20 data points can be collected in just over four minutes. This represents a dramatic reduction in testing time.

### Training Session

A training session was employed in order to familiarize participants with their task and to tune the model to each participant. The training session involved a small 2D Gaussian luminance “blob” (a 2D Gaussian luminance profile) positioned on the outer rim of the circular stimulus area performing a random walk. The walk consisted of stepping $10^{\circ }$ either clockwise or anticlockwise with equal probability, every five seconds.

The goal of the participant was to direct the target’s motion towards the center of the blob. As we wanted our participants to get used to a moving background, during the training sessions we had each randomly oriented background Gabor element move at a random speed creating a sense of movement in the background but without any coherent global direction.

The target direction was varied by the participant using a mouse where rightward movement produced a clockwise change in direction and leftward movement an anticlockwise change. Training sessions lasted 130 seconds but the first 10 seconds of data was not analyzed.

**Figure 5.**
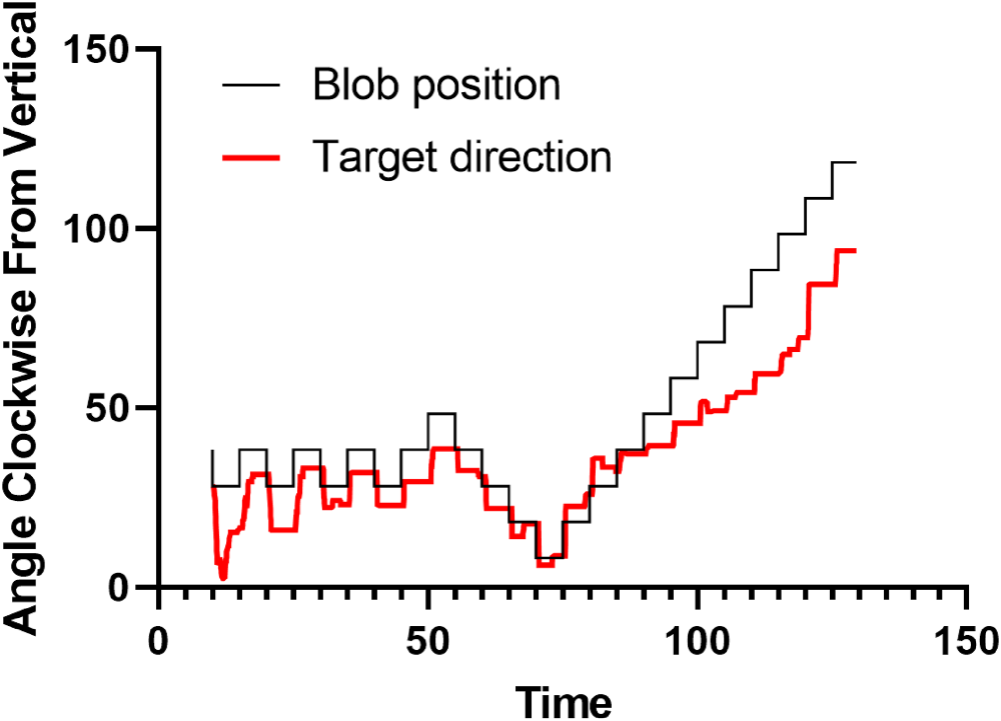
Example time series for blob angular position and target direction during a training session. The participant’s task was to adjust the target direction so that it appeared to move towards the blob which was positioned 17 degrees from fixation outside of the background display area. Participant ED.

Figure 5 shows the last 120 seconds of an example time series for the computer-generated blob direction and participant-driven target direction for participant ED.

As expected, the target data lags behind the blob data, the transitions lack the same square step profile as the blob steps, and the steps end on final values that are some distance from the actual blob values. The average time lag for a response was 0.41s and the variance of the difference between the blob and target data at the end of each blob direction step was $1.6\;\deg^{2}$ . These constitute estimates of Δt and $\sigma_{\omega }^{2}$ ( $=\sigma_{\psi }^{2}=\sigma_{\phi }^{2}$ ), respectively (see Figure 3 and equations A.12, A.2, A.8, and A.11 respectively).

**Figure 6.**
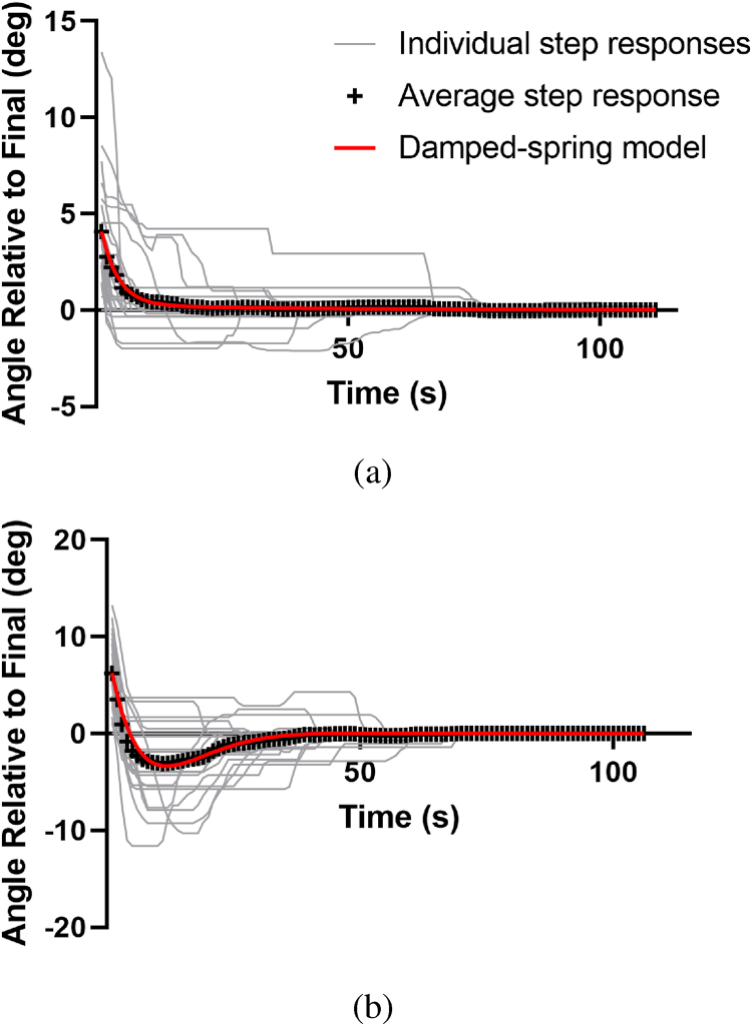
Rectified responses to steps for two participants. Top: Target direction values following the blob steps depicted in Figure 4 for participant ED. Individual responses to a blob step (light gray), the average response (+) and the damped spring model (red) are shown. Bottom: Equivalent step responses for participant MF for comparison. MF’s optimal spring model has a relatively lower damping constant leading to the overshooting seen in the figure.

To get a sense of how participants responded on average to a single blob step, the responses to each blob step were aligned, rectified to make all steps in the same direction for comparison, and averaged. Figure 6 shows individual rectified responses to steps, the average step response and the result of fitting the damped-spring model in the last panel of Figure 3 (equation A.13) to the average step response for two participants.

The optimal spring constants for ED were b=0.375 and c=0.008 . For comparison, they were b=0.272 and c=0.024 for participant MF representing a stronger spring with less damping. The expectation is that these spring characteristics will persist for each individual in the main experiment so these constants were applied when analyzing experimental data.

The MATLAB code used to analyze the training data is included in Supplemental Materials 3.

### Experimental Session

The only differences between the experimental and training stimuli were that the Gaussian blob was not present for the experiment and the background elements drifted coherently to produce a sense of movement in a single direction, just as was done with the target. Target and background speeds were equal but directions were different. The background direction is the independent variable and was controlled by the computer while the target direction is the dependent variable and was controlled by the participant using a mouse as in the training session. Sessions lasted 130 seconds but the first 10 seconds were not analyzed as with the training session. To ensure that the background direction walk was truly random while also making sure it covered the direction angles under investigation, before each session the program was used to generate a series of trial random walks. The first of these that covered the desired direction space was selected for use. To match the previously conducted trial-based experiment our desired background direction range was the top right quadrant of Euclidean space i.e. 0∘–90∘ measured clockwise from vertical, plus a 10∘ buffer on either side.

**Figure 7.**
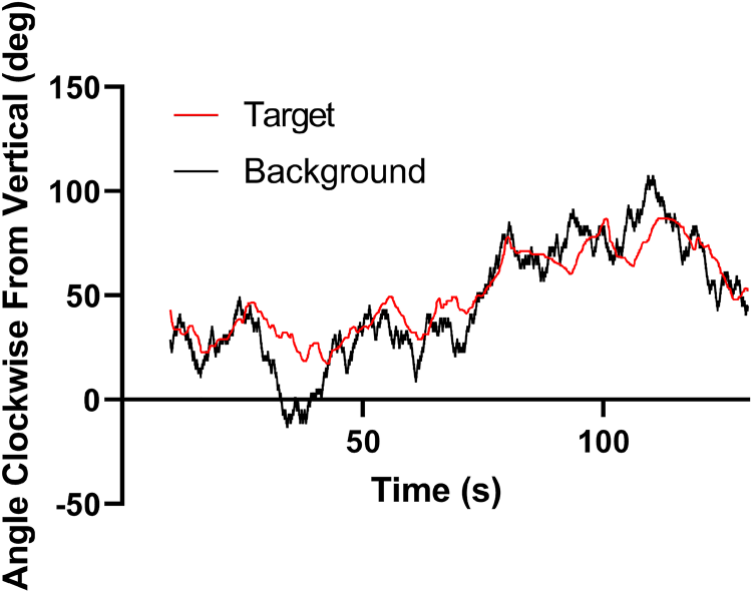
Example time series for background and target directions. The background direction ( x ) underwent a random walk under the control of the computer while the participant adjusted the direction of the target ( y ) so that it appeared to move vertically upwards (equations A.1 and A.7 describe the processes for the generation of x and y data). Participant ED.

Example raw time series for the target and background directions are shown for participant ED in Figure 7.

### Analysis

The relationship between x , y and perceived y depicted in Figure 7 is obscured by noise resulting from the use of a continuous regime. These three variables are all related by the theoretical function $f(\hat{x},\hat{y})$ depicted in the perception panel of Figure 3 and described in equation A.11. This function operates on processed versions of x and y denoted $\hat{x}$ and $\hat{y}$ and produces perceived y that are typically not ideal (i.e. vertical) because time is not allowed at each iteration for the participant to entirely correct for current deviations from vertical. Using the BP model we can calculate new x and y data sets that are closer to those that are actually experienced at the perceptual stage and we can correct for deviations of the perceived target from vertical.

We denote the corrected data sets $x^{*}$ and $y^{*}$ . $x^{*}$ is simply $\hat{x}$ as given by equation B.1. $y^{*}$ is $\hat{y}$ corrected for deviations of perceived y from vertical i.e. $y^{*}=\hat{y}-\tilde{y}$ . Recall that $\tilde{y}$ represents the deviation of perceived y from goal y as described by equation B.2. An explanation for the generation of the new data sets $x^{*}$ and $y^{*}$ is given in Appendix B.

**Figure 8.**
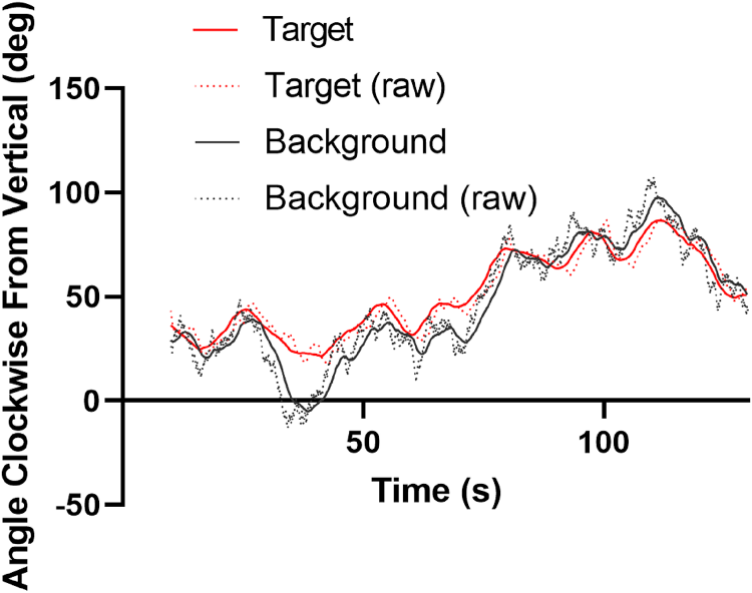
Final data after analysis. Time series for target and background (solid lines) along with raw versions of the same (dotted lines taken from Figure 7) for comparison. The solid black line depicts a time series for $x^{*}$ and the red a time series for $y^{*}$ (see equations B.1 and B.2).

We would expect to see a decreased variance in the xy relationship and a clearer systematic relationship between x and y if our analysis is aligned with the *actual* processes occurring in real participants. The time series for $x^{*}$ and $y^{*}$ is shown in Figure 8.

The new background time series is a smoother, lagged version of the original. The new target time series is predominantly an estimate of the intended damped spring-like component of the participant’s actions minus the estimated deviations of the perceived target direction from vertical. The *intended* component because the unintended action noise $\lambda_{t}$ is multiplied by $k_{y}$ as per equation A.8 which, in this case, was 0.00052 . This essentially slides the target time series backwards by a small amount and smooths any sharp features.

For this particular data set the action noise variance ( $\sigma_{\lambda }^{2}$ in equation A.7) is $0.22\;\deg^{2}$ , the time lag introduced by the Bayesian background estimation process ( Δt in equation A.12) is 1.51s , the time lag introduced by the damped-spring action system ( $\Delta t^{\prime }$ described in Appendix B) is 2.01 s and the remaining time lag between features in the target and background time series ( $\Delta t^{*}$ described in Appendix B) is zero. This means that lags between the original background and target data are fully accounted for by a sluggish representation of the independent background direction due to uncertainty about its direction changes, the time taken to react to the background change and sluggishness in the action system due to its spring-like nature.

**Figure 9.**
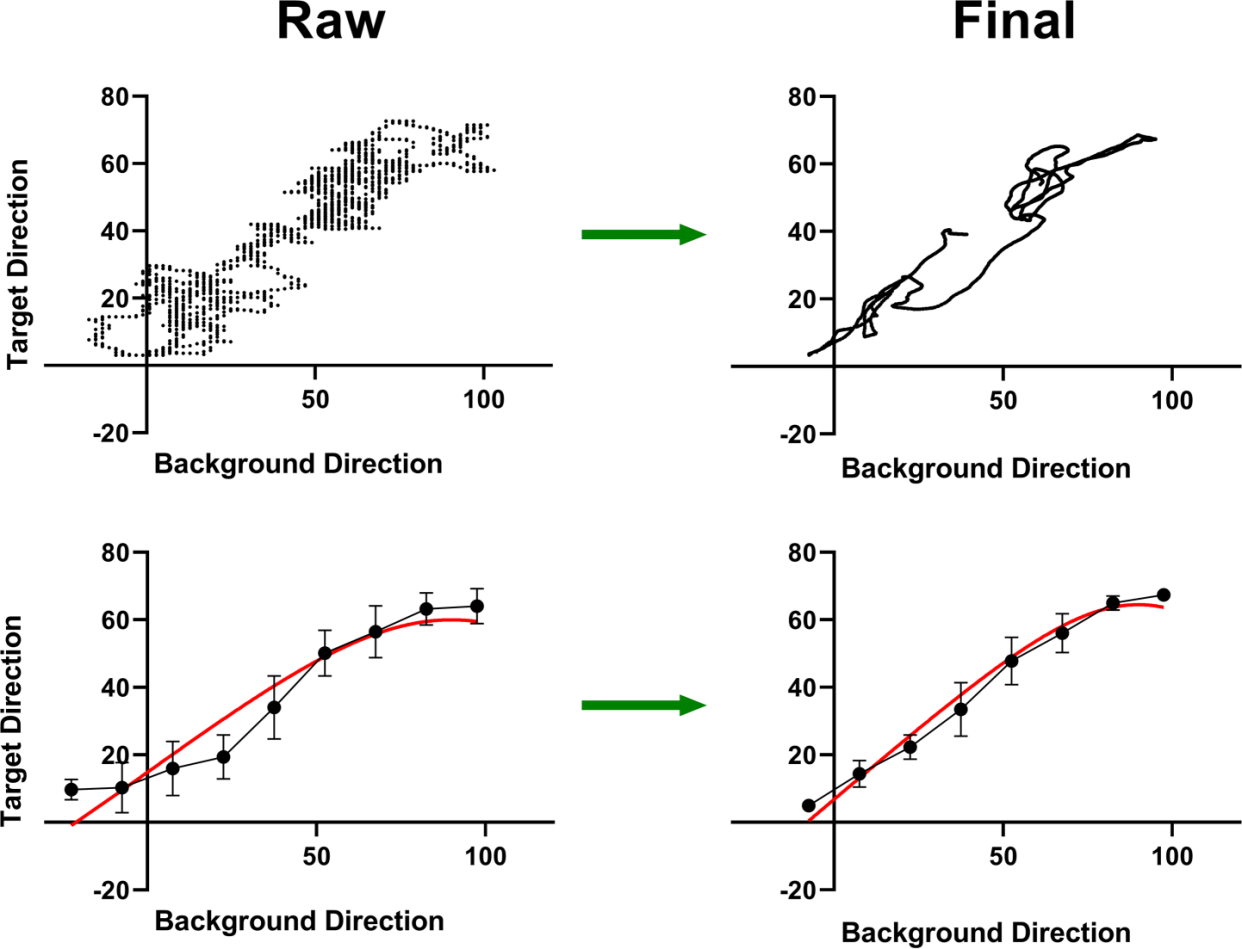
Raw (x and y) and Final $(x^{*}$ and $y^{*})$ data plotted as target direction against background direction. Top: Scatter plot. Bottom: Background direction range divided into nine bins with averages and standard deviations plotted for those bins. Red lines are fits of the model expressed in equation 1. Same data as that shown in Figure 7 and Figure 8 for participant ED.

In Figure 9 we plot in two ways raw y , representing the target deviation from vertical, against raw x , representing the background direction relative to vertical. In the first case, we show a scatter plot of the data. The second plot is a binning of the data into nine evenly distributed bins with bin number chosen to approximate background directions in our trial-based version of the experiment. We compare these plots to the equivalent plots for $x^{*}$ and $y^{*}$ in the same figure.

Comparing the raw x versus raw y scatterplot with final $x^{*}$ versus $y^{*}$ in the top row of Figure 9, we see that:

- The general trend in target direction as a function of background direction does not change appreciably as a result of analysis but does change significantly at a local level. For example, where it is impossible to trace target changes resulting from background changes in the raw data it is very clear in the processed data. In the processed data, fields of data points give way to distinct lines representing target values as a function of background values.
- For the background directions that occur more than once, the corresponding target directions are generally more similar after analysis. Analysis reveals a consistency in target direction choices for a given background direction that is not obvious in the raw data. The close-to-overlapping pairs of lines at the lower and upper ends of the curves for the final data are clear examples.

Consistent with the last point, the average of the standard deviations represented by the error bars in the bin plots on the bottom row of Figure 9 decreased from $6.52^{\circ }$ to $4.05^{\circ }$ . A fit of the model to the data is shown in red and describes well the relationship between target direction and background direction (Falconbridge et al., 2022). This is our best guess at the function f and is denoted $f^{*}$ . Our model of the horizontal component of perceived y relative to vertical is represented by:

(1) $${\tilde{y}}_{x}^{*}=f_{x}^{*}(x_{x},y_{x})=y_{x}-\beta x_{x}+\cos\;d$$

Here $x_{x}$ and $y_{x}$ are the horizontal components of the vectors representing background and target motion respectively and d is a free parameter added to allow for any constant offset a participant may have in their target direction settings relative to background direction. Such an offset results in a nonzero y -intercept as seen in Figure 9.

The quality of data fit provides a measure of the strength of a systematic relationship between raw x and y as well as between final $x^{*}$ and $y^{*}$ . For the former, the mean square distance between data and model was $27.6^{\circ }$ and for the latter, it was $3\times 10^{-6\circ }$ as determined by using GraphPad Prism’s V9.0.2 least squares nonlinear regression method. Two other measures of how well the data is described by the model are $R^{2}$ which measures how much of the data variance can be accounted for by the model and Akaike’s Information Criterion (AIC) value which is a function of the likelihood that the model represents the underlying cause of the data. $R^{2}$ rose from 0.92 to 0.98 and AIC value fell from 43.3 to 29.6 which represents a highly significant improvement (a change of 2 is considered significant, see (Burnham & Anderson, 2002)).

This overall improvement was consistent for all data sets analyzed by us. For the participants ED, MF, and CB whose data is presented below, the average reduction in target standard deviation per bin was significant at $-2.13^{\circ }$ ( t (8) = 3.70 , p = .003 , one-tailed paired t-test). The mean square difference from the model decreased significantly from an average of 11.94 to 0.00014 ( t (8) = 2.30 , p = .025 , one-tailed paired t -test). The average $R^{2}$ went from 0.70 to 0.90 which is a significant change ( t (8) = 2.31 , p = .05 , paired t -test) and AIC average value fell from 39.0 to 33.4 which represents a highly significant improvement.

The MATLAB code used to analyze the experimental data is included in Supplemental Materials 2.

## Comparing Results to Trial-Based Results

An important measure of any new method is how well its results compare to those from previous established methods. To that end, we compare the results of the continuous experiment just outlined to those from a trial-based version of the same experiment conducted in our lab. We present data from individuals who participated in both experiments.

**Figure 10.**
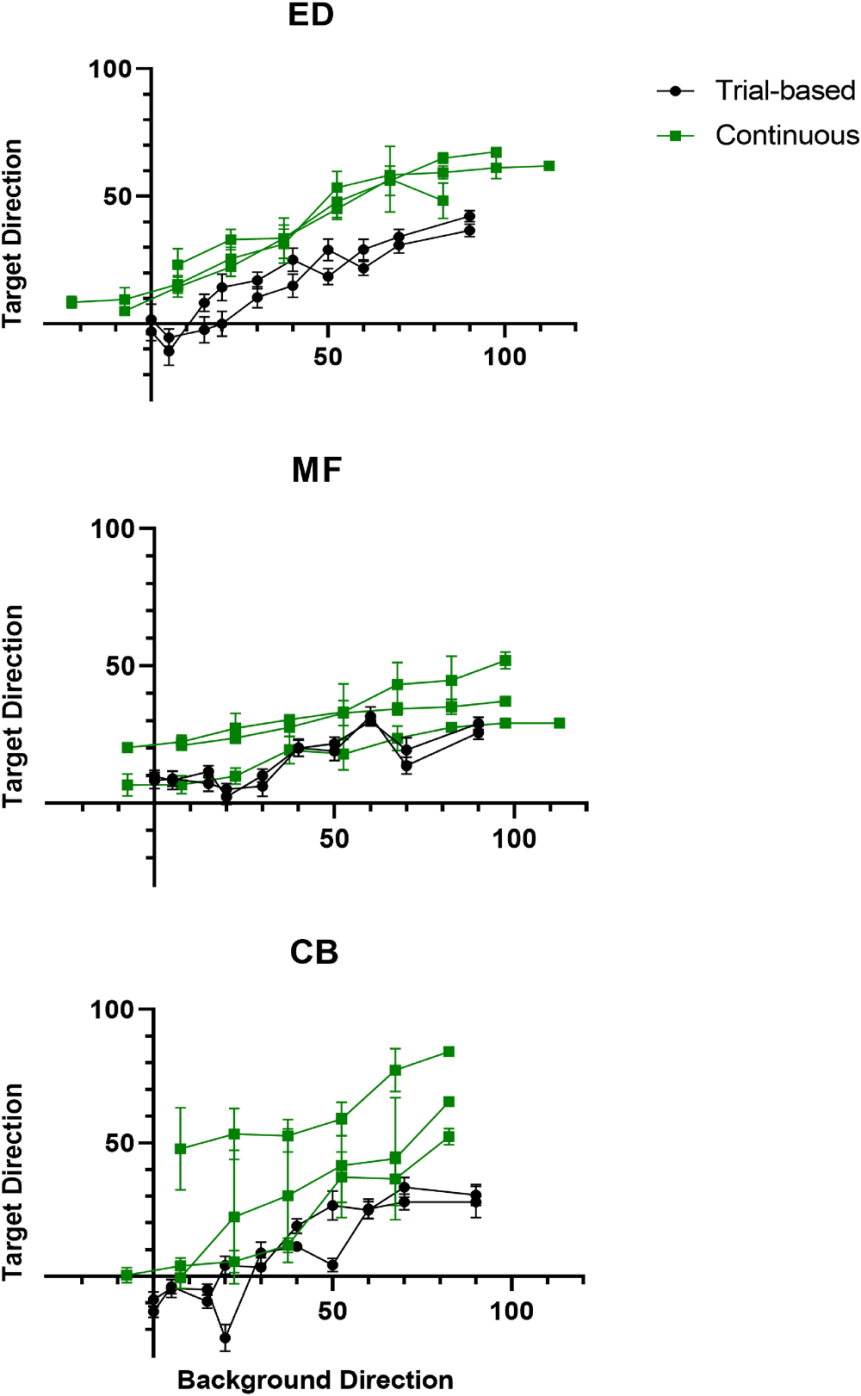
Continuous compared with trial-based data.

The trial-based version of the experiment is described in (Falconbridge et al., 2022). For each session, a background motion direction was randomly selected from the set represented in Figure 10. For each trial, a target direction was selected and the resulting stimulus displayed for 0.5 seconds. The observer responded with a button press indicating whether the target appeared to be moving in a direction left or right of vertical. The Psi-Psychophysical Method built into the Palamedes MATLAB toolbox (Prins & Kingdom, 2018) was used to determine which target direction to display on the next trial based on previous responses. This method is considered efficient for trial-based approaches and produced an estimate after 75 trials of the target direction that most likely appeared vertical for the observer for the given background direction along with an estimate of the error in the prediction as depicted by the error bars in the plot. Each session took about 10 minutes to complete making the data collection time for each curve about 1 h and 40 min.

The first thing to note is that the slopes are similar between experiment types for each individual in that the change in target direction for a given change in background direction is similar between experiment types. This aspect of the relationship was measured using the parameter β , representing the strength of the induced motion effect, in our model. The mean β values for the trial-based experiment were 0.67 , 0.35 , and 0.70 for observers ED, MF, and CB, respectively. The equivalent means for the continuous experiment were similar at 0.71 , 0.37 , and 0.76 . Matched paired t -tests showed no significant differences (individual values and group values). Note that the large variations in β value between individuals seen here appear to be typical for the induced motion effect (Falconbridge et al., 2022; Zivotofsky, 2004) and may result from differences in preference for assigning background motion to self-motion while actually being stationary (Falconbridge et al., 2022; Warren and Rushton, 2007) which may vary by experience.

Unlike the slopes which are consistent for each individual, there is a clear difference between the average vertical offsets in Figure 9 for the two experiment types, particularly for participant CB. The offset is represented by parameter d in our model which was optimized during model fitting. The average offsets were $13.5^{\circ }\pm 15.1^{\circ }$ (95% CI), $14.2^{\circ }\pm 18.8^{\circ }$ and $10.0^{\circ }\pm 58.5^{\circ }$ for ED, MF, and CB, respectively. For the group the average offset was $12.6^{\circ }\pm 9.9^{\circ }$ which is significantly different from $0^{\circ }$ ( t (2) = 9.67 , p = 0.011 , one-sample t -test).

The cause of the positive offset may have been adaptation. For the 130s duration of the data-collecting session, the participants viewed target and background motion that almost always had a positive rightward component. Desensitization to rightward motion due to prolonged exposure may have caused the participants to adjust the target more clockwise of vertical as the experiment progressed.

All participants reported at the end of each session when all motion in the scene had ceased and the target and background elements remained on the screen for a second or so, that the target appeared to drift to the left (and downwards). This was noteworthy because, in accordance with the aim of the participant, the prevailing perception during the session was that the target was drifting vertically upwards, not to the right, so a motion after-effect that was leftward was not expected.

In terms of our BP model this indicates that adaptation was occurring at the “sensation” stage of (at least) the dependent variable processing stream where the actual motion of the target (which had a strong rightward component on average), not the perceived motion (which did not), is estimated by the brain. Adaptation at this level would result in progressively lower sensitivity to rightward target motion and, to compensate, one would expect participants to adjust the target in a more and more clockwise direction which would lead to the positive offset observed.

Note also that the reported leftward aftereffect was *relative* to the background. Although participants may have adapted to the rightward component of the background over time which would make the background less effective at repulsing target motions which, in turn, would potentially counteract adaptation to the target motion, this effect was not as strong as the target adaptation effect.

### A Symmetric Version of the Continuous Experiment

**Figure 11.**
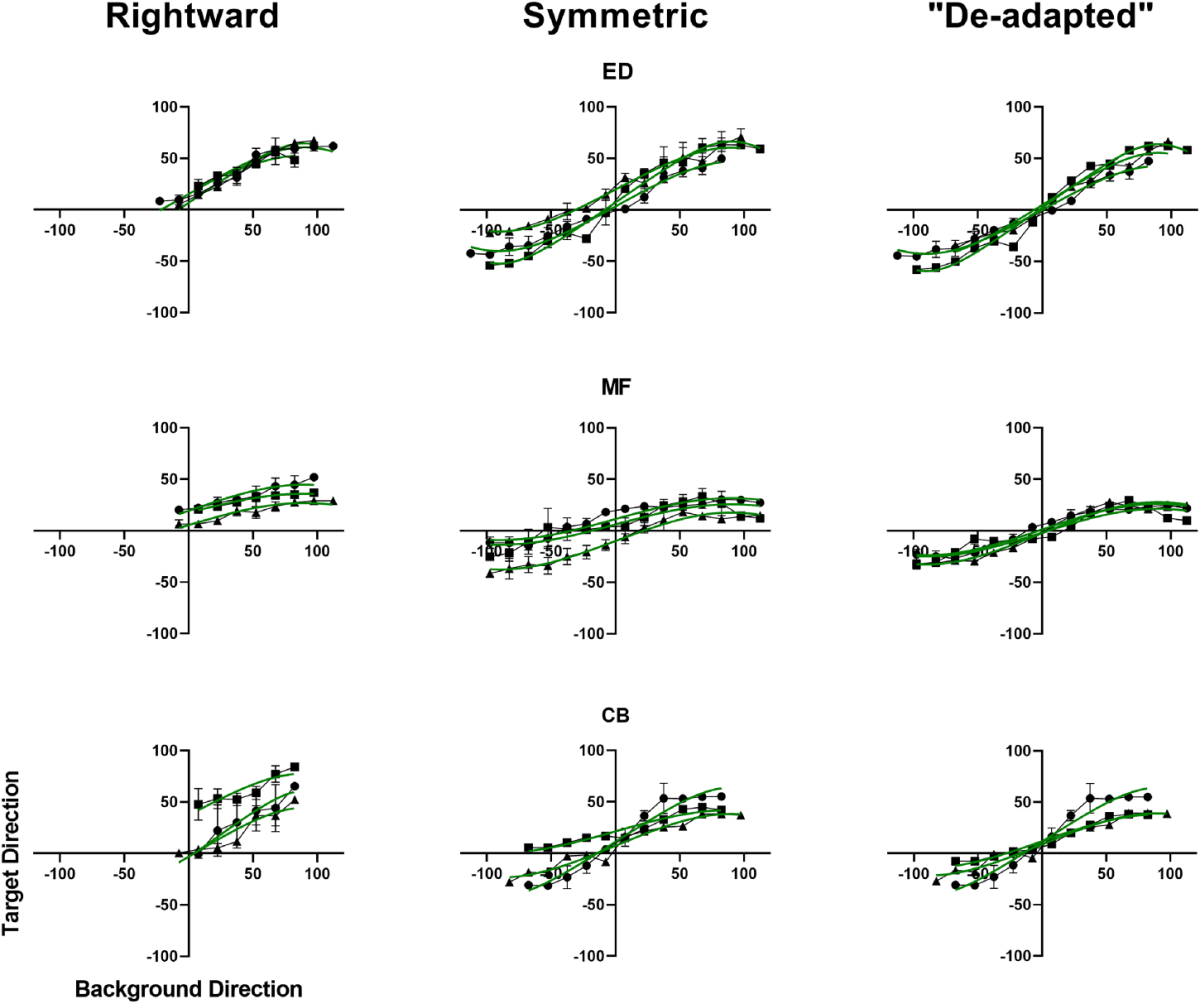
Data from Rightward and Symmetric experiments compared along with a “De-adapted” version of the symmetric data.

In an attempt to avoid the target adaptation effect, we conducted a second experiment where the random direction walk of the background spanned a full 180∘ centered on vertical so that there was, on average, an equal amount of leftward and rightward motion for the background, and by extension, the target. In order to cover more ground without the background having to step faster we extended the data collection time from 120 seconds to 240 seconds making each session 250 seconds long. The resulting data and model fits are shown in Figure 11. Data and model fits for the first rightward motion-containing experiment are shown for comparison in the left column. In the right column are shown “de-adapted” versions of the same data where a simple adaptation model has been applied. This model is explained in the next subsection. All plots use the same axes for ease of comparison.

Interestingly, the offset persisted in the symmetric version of the experiment. It dropped from an average value of $12.6^{\circ }$ to a value of $8.6^{\circ }\pm 7.1^{\circ }$ but the offset remained significantly different from zero for the group ( t (8) = 2.81 , p = 0.023 , one-sample t -test). Nevertheless, the persistent offset may still be a result of adaptation. For all participants, the data sets with the higher offsets came from sessions where the random background and target directions happened to be almost exclusively clockwise of vertical for the first half of the session. This was the case for 5 out of 9 sessions. This equates to about two minutes of rightward target motion, just like in the original rightward experiment before there is any leftward motion.

By the adaptation hypothesis, this would lead to an accumulated desensitization to rightward motion and thus a shifting of target directions more clockwise. When the background passes from positive to negative values the target values would be biased in a positive direction. The bias may slowly disappear during the second half of the experiment but the average target directions would be more clockwise than expected.

Only 3 of the 9 sessions happened to have almost exclusively leftward background motion in the first half and, consistent with our hypothesis, these produced the most negative offsets. The other two sessions had a mix in the first half and the resulting offsets were in the middle of the group. It should be possible to minimize these effects by a more deliberate selection of the set of background motions in the testing sequence.

### Simple Adaptation Model

To test the adaptation hypothesis further we applied a very simple adaptation model to the symmetric data. The model asserts that the longer the exposure to a rightward (leftward) component of motion and the greater the magnitude of the rightward (leftward) component, the more the user adjusts the target to the right (left) in order to counteract a decreased sensitivity to rightward (leftward) motion. To that end, for each target data point like those shown in Figure 7, we subtracted a constant q times the horizontal component values for all previous time steps to create a new data set devoid of the adaptation effect. The constant q was optimized for each data set to produce “de-adapted” target x-components that had regression lines passing as close as possible through the origin using MATLAB’s fminsearch algorithm. Average q values for ED, MF, and CB were $4.1\times 10^{-5}$ , $1.9\times 10^{-4}$ and $7.8\times 10^{-5}$ respectively.

The resulting data sets are represented in the right column of Figure 10 for participants ED and MF. The resulting average offset for the group was $1.0^{\circ }\pm 1.8^{\circ }$ which is not significantly different from $0^{\circ }$ ( t (8) = 1.25 , p = 0.25 , one-sample t -test). This should not be surprising as the q values are deliberately chosen to decrease the offset in the target so that the value of applying the adaptation model must be measured in other ways.

As with our BP model, we ask whether the application of the adaptation model produces a more reliable relationship between the background and target data. For ED and MF it does, but for participant CB it does not. The data shown in the bottom left and middle plots for CB in Figure 11 suggests a clockwise bias in the perception of vertical. Accordingly, rather than optimizing the adaptation constant so that the regression line passed through the origin, we optimized for a positive y -intercept. The optimization producing the best relationship between the target and background data has an offset of $9^{\circ }$ representing a potential bias in CB’s perception of vertical that is $9^{\circ }$ clockwise of vertical. The resulting target/background relationship is plotted in the bottom right of Figure 11.

Note that the positive bias is not apparent in CB’s trial-based results in Figure 10 indicating that there was something about the stimulus, task, and/or other factors in the continuous experiments that evoked the bias. As the trial-based data was collected two years previously it is also possible that the bias developed via external factors over time.

As indicated by the curves and error bars in the last column of Figure 11, applying the adaptation model increased the reliability of the relationship between the target and the background data. The average $R^{2}$ value rose from 0.94 to 0.96 (nonsignificant, t (8) = 1.98 , p=.09 , paired t -test) and the average AIC fell from 52.2 to 49.7 which represents a significant improvement in the predictive power of the model. At the same time, average variation in target values for each given background value, as represented by the error bars in Figure 10, also decreased significantly from 5.57 to 5.32 ( t (120) = 2.51 , p=.01 , paired t -test). This represents an overall improvement in data statistics by the addition of the adaptation model indicating that it likely reflects that the participants were actually experiencing adaptation, as would be expected from periods of continuous exposure to a moving pattern. In the General Discussion, we consider the validity of the adaptation rates, q , that were used to produce our fits.

Note that average β values were not significantly altered for each individual by the switch to a symmetric regime ( t (4) = 0.25 , 0.24 and 0.25 ; p=.81 , .82 and .81 for ED, MF, and CB, respectively; two-sample t -test) nor by the addition of our adaptation model ( t(4)=0.69 , 1.52 and 0.11 ; p=.53 , .20 and .92 ; two sample t -test comparing rightward to de-adapted data).

## General Discussion

We have demonstrated, using a continuous version of an induced motion experiment, that our BP model can be effectively applied to analyze data from continuous psychophysics experiments. It allows the conversion of noisy raw independent and dependent variable data sets into cleaner data sets that are comparable to those from traditional trial-based experiments. Table 1 lists the the “pros” and “cons” of our continuous approach compared with a traditional trial-based approach based on our direct experience with the two.

**Table 1.** The advantages and disadvantages of our continuous approach compared to our trial-based approach.

Advantages	Disadvantages
Data collection is rapid	There is a need for a training session
Task is engaging	Task can feel “intense”
Natural perception/action loops engaged	The possibility of adaptation

In converting from the raw continuous data to our “ideal” data using our BP model, the amount of variance in the data significantly decreased and the relationship between the independent and dependent variables became significantly clearer bringing the data closer to that from a more controlled trial-based experiment. If target perception truly is a function the actual target and background motions, then there should be a consistent relationship between those three variables if noise sources can be removed. The application of a good model of noise sources inherent in the perceptual and action systems should reveal the consistent relationship whereas an incorrect model would further disguise the relationship.

There is decreased variability in the final data produced using our BP model as well as a more consistent relationship between perceived and actual target/background motions. This indicates that the principles upon which the BP analysis is based are a good approximation to the true perception and action principles active in real participants in a continuous experimental environment.

As technology improves, the possibility of collecting human behavioral data rapidly and under more naturalistic conditions becomes increasingly viable. In order to make use of such data, new models of the relationship between internal psychological states and outward stimuli and actions under continuous, dynamic experimental conditions need to be proposed and validated. Our BP model represents such an attempt.

It is a model, specifically, of a participant’s internal states and actions in the case of an above-threshold, continuous-correction two-variable experiment, where one variable is continuously adjusted by the participant in response to continuous changes in a second variable that influences the first. In Appendix D we show that it is also possible to apply the model to continuous *tracking* experiments involving two variables. We have argued that studies looking at relationships between two above-threshold variables are ideal places to apply a continuous approach.

The data analysis approach taken in our study was to take the dependent and independent data produced by the participant and computer respectively and to refine it. Refining equates to calculating the inputs and outputs of an imagined function f that represents the mechanism driving the interaction between the dependent and independent variables. The inputs are Bayes-optimal estimates of the dependent and independent values assuming an agent whose visual system receives noisy copies of the raw data. The outputs are estimates of dependent “perceptions” that drive the actions of the participant.

We have shown in the main text and Appendix A how the perceptual system that creates the inputs to f is Bayes-optimal. We have only briefly discussed how the action system is also Bayes-optimal. For the interested reader, in Appendix C we show how the action system is consistent with BDT (Parmigiani et al., 2001).

### Adaptation Issue

Implementing our BP model with our induced motion continuous experiment revealed a potentially disruptive issue with the continuous psychophysics regime, namely the issue of adaptation. Stimulus exposure times in continuous experiments are generally much longer than they are for trial-based experiments and years of research have established that perceptual systems turn down the gain on representations of persistent stimuli (Webster 2015).

In our case, we had motion in a persistent direction which appeared to reduce sensitivity to motion in that direction leading to participants increasing the motion component in that direction. What our experiment also highlights is that the adapting component of the stimulus may not be registered consciously. Our participants behaved, in our first experiment, as if adapting to a persistent rightward component of target motion, but consciously they would have seen the target moving predominantly upwards, as their task was to keep perceived target direction vertical. Our model offers the explanation that the adaptation must have been occurring at a pre-perceptual stage of processing (sensation stage in Figure 3) where rightward target motion would have been registered.

We offer two approaches for dealing with adaptation in continuous experiments. The first is to alter the stimulus to avoid longer-than-needed periods of exposure to persistent stimulus attributes. This reduced adaptation effects in our case but it did not erase them completely. Our results suggest that a more deliberate selection of the independent variable random walk may further reduce adaptation effects.

The second approach is to remove adaptation effects from the data with the use of a simple adaptation model. We do this in the present paper by simply subtracting a small constant times the sum of the horizontal components of all previous dependent data points. We showed that the resulting data was improved by the addition of this analysis step indicating that our model reflected a process that was actually occurring. Evidence for the process is apparent by consistency in the data and the fact that the difference between the expected relationship between variables and the measured data decreases as a result of applying our adaptation model.

Our simple model of adaptation postulates that at any time, the decrease in sensitivity to rightward positive or negative target motion is simply a constant times the amount of rightward motion accumulated up until that moment i.e. $\sum_{\tau =0}^{t}y_{\tau }$ . However, motion adaptation studies show that the time course of adaptation to a constant motion stimulus is exponential in nature. Sensitivity decreases quickly early on and more slowly as exposure time progresses (Bex et al., 1999; Giaschi et al., 1993; Hammett et al., 2000). The difference for us is that our motion directions changed with time, thereby stimulating new populations of direction-selective cells (Albright, 1984; Hubel & Wiesel, 1959; Zeki, 1978) as significant direction changes randomly occurred.

A given set of neurons may have adapted for a while, recovered, then adapted again as the target direction realigned with the cells’ preferred direction. Our analysis found that the accumulated effect was better modeled by a linear function than an exponential one. The average constant q value was 1.0 x 10^-4^ which would mean that if the target were moving directly to the right at 6∘/s (the actual target speed in our experiments) its perceived speed after 1 s would drop to 5.988∘/s thereby changing by a factor of 0.2%. If this perceived speed change is extrapolated, it would be about half that found over a two-minute adaptation period to a similar speed grating moving at a constant velocity in Thompson (1981) and a 30s adaptation period to a similar stimulus in Bex et al. (1999). This appears plausible given the diffuse allocation of adaptation amongst different populations of neurons over time in our experiment.

### Relationship to Other Continuous Psychophysics Approaches

Bonnen’s (2015) Kalman filtering approach and Straub’s (2022) Linear Quadratic Gaussian control approach to analyzing data from continuous experiments are closest to our approach. These studies are the only continuous psychophsyics studies among those cited in the Introduction where a dynamic model of a participant in a continuous environment is attempted. Bonnen’s is a model of just the perception side of the participant (using a Kalman filter approach) whereas Straub’s is composed of a Kalman filter which models perception and (various forms of) a Linear Quadratic Regulator which models the action system of the participant. Both models are designed to deal with data from single-variable continuous *tracking* experiments where participants are tasked with tracking just a single stimulus variable, for example, the position of the center of a random-walking Gaussian blob. In relation to our model, this represents a single-stage, single-variable model equivalent to just the two top left panels of Figure 3 in the case of Bonnen’s model and the two top left panels plus the bottom right panel in the case of Straub’s. In theory, it should be possible to extend these models to deal with two variables. In comparison to Straub’s, our model is much simpler in that it has fewer equations and parameters for the same aspects of the model which makes the modeling of more complex experiments more tractable. However, it also means that fewer internal states can be tracked.

As it is, our BP modeling approach represents a major step forward for continuous psychophysics as it offers a possible picture of the internal states of a participant in an above-threshold, two-variable continuous experiment and allows for the analysis of data from such experiments; both continuous correction and continuous tracking-type experiments. As argued above, quickly exploring the relationships between two above-threshold stimulus variables is an ideal application for continuous psychophysics.

## Supplemental Material

sj-m-1-ipe-10.1177_20416695231214440 - Supplemental material for Continuous psychophysics for two-variable experiments; A new “Bayesian participant” approach Supplemental material, sj-m-1-ipe-10.1177_20416695231214440 for Continuous psychophysics for two-variable experiments; A new “Bayesian participant” approach by Michael Falconbridge, Robert L. Stamps, Mark Edwards and David R. Badcock in i-Perception

sj-m-2-ipe-10.1177_20416695231214440 - Supplemental material for Continuous psychophysics for two-variable experiments; A new “Bayesian participant” approach Supplemental material, sj-m-2-ipe-10.1177_20416695231214440 for Continuous psychophysics for two-variable experiments; A new “Bayesian participant” approach by Michael Falconbridge, Robert L. Stamps, Mark Edwards and David R. Badcock in i-Perception

**Figure:** Video 1:

## Figures and Tables

**Figure 12.**
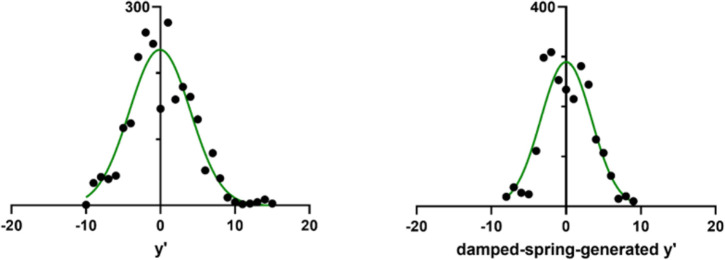
Histogram of $\tilde{y}$ values driving changes to y for participant ED (left graph) compared with damped-spring-generated $\tilde{y}$ values (right graph). The first distribution is taken to reflect the utility of $\tilde{y}$ values and the second to reflect the probability of $\tilde{y}$ values under a damped-spring action policy. Expected utility is high when the profiles of the two distributions match. Also included are the best-fitting (nonlinear regression) Gaussians for comparison (green lines).

## Appendix A

### BP Model

To set up the BP model, knowledge of the dynamics of the independent component of the stimulus and a sense of how the participant acts on the dependent part of the stimulus is required. We have assumed in this study that the independent stimulus component, which is the part of the stimulus that is not controlled by the participant, is perturbed randomly at each iteration. For the action system, we have assumed damped-spring-like behavior. Below we lay out the BP model that follows from these assumptions beginning with those parts related to the independent variable followed by the dependent variable, their interaction and ending with the action system.

Note that we have assumed Markovian processes throughout the system—both in the world and in the BP, that is, current states rely only on immediately preceding states. Thus, all update equations are of the form $j_{t}=j_{t-1}+\cdots$ where j might represent an internal (to the participant) or external state.

We have also assumed *unbiased* estimates of world states. It may well be that in humans, past experience or genetics have endowed the visual system with biases in the priors, but we start, here, with the simplest case; that priors emerge from the experimental data alone.

In the equations to follow, lower case letters refer to world states (e.g. x ), upper case letters refer to low-level sensory representations of those states (e.g. X ), hats indicate that the variable is an internal estimate of a world state (e.g. $\hat{x}$ ), and a tilde indicates a higher-level “perceived” representation of a world state (e.g. $\tilde{y}$ ). Greek letters indicate additive Gaussian noise.

#### The Independent Variable, x

The independent variable—the background direction in our study—is randomly perturbed at each iteration so that participants can not guess where it will go next.

(A.1)
$$x_{t}=x_{t-1}+\mu_{t}\;\;\;\;\;\;\;\;\;\;\;\;\;\;\;\;\;\;\;\;\mu_{t}∼N(0,\sigma_{\mu }^{2})$$

The transduction of x into a sensory representation of that variable simply involves the addition of noise.

(A.2)
$$X_{t}=x_{t}+\omega_{t}\;\;\;\;\;\;\;\;\;\;\;\;\;\;\;\;\;\;\;\;\omega_{t}∼N(0,\sigma_{\omega }^{2})$$

It is assumed that the BP possesses a “generative” model; an internal model of the external stimulus generative process. To be clear, we distinguish the internal from the external variable, x , with the use of a hat, that is, by use of the symbol $\hat{x}$ . The value of $\hat{x}$ at time t is obtained by estimating the most likely cause of the sensory data, $X_{t}$ , that is, by setting ${\hat{x}}_{t}$ to the peak of the posterior distribution $p({\hat{x}}_{t}|X_{t})$ which, by Bayes’ formula is

(A.3)
$$p({\hat{x}}_{t}|X_{t})=\frac{\;p(X_{t}|{\hat{x}}_{t})p({\hat{x}}_{t})}{p(X_{t})}.$$

As we are only interested in the position of the peak of this distribution in ${\hat{x}}_{t}$ space for a given $X_{t}$ and not the value of this peak, the denominator on the r.h.s. is ignored. Based on equations A.1 and A.2, $p(X_{t}|{\hat{x}}_{t})$ has a mean of $X_{t}$ with a variance of $\sigma_{\omega }^{2}$ in ${\hat{x}}_{t}$ space and $p({\hat{x}}_{t})$ has a mean of ${\hat{x}}_{t-1}$ and a variance of ${\hat{\sigma }}_{\mu }^{2}$ in that same space. Note that ${\hat{\sigma }}_{\mu }^{2}$ is an internal estimate of the external variance $\sigma_{\mu }^{2}$ . All distributions are normal making the numerator of equation A.3

(A.4)
$$p(X_{t}|{\hat{x}}_{t})p({\hat{x}}_{t})=g({\hat{x}}_{t};X_{t},\sigma_{\omega }^{2})g({\hat{x}}_{t};{\hat{x}}_{t-1},{\hat{\sigma }}_{\mu }^{2})$$

where

(A.5)
$$g(a;b,c)=\frac{1}{\sqrt{2\pi c}}\exp\left(-\frac{(a-b{)}^{2}}{2c}\right).$$

Multiplication of the two distributions produces a Gaussian with a mean of

(A.6)
$${\hat{x}}_{t}=(1-k_{x}){\hat{x}}_{t-1}+k_{x}X_{t}\;\;\;\;\;\mathrm{where}\;\;\;k_{x}=\frac{{\hat{\sigma }}_{\mu }^{2}}{{\hat{\sigma }}_{\mu }^{2}+\sigma_{\omega }^{2}}.$$

just as in Figure 3.

This *is*, indeed, the equation describing the update of $\hat{x}$ for the BPmodel, but as experimenters, we do not have access to the internal state $X_{t}$ . This makes the determination of the $\hat{x}$ time series for the purpose of data analysis impossible. Fortunately, we can approximate $X_{t}$ with $x_{t}$ —the raw data equivalent of this variable. This is because, according to equation A.2, one is just a noisy version of the other; they will match one another on average. Nor do we have access to the internal estimate of the variance in μ . Again, an experimenter can approximate the internal ${\hat{\sigma }}_{\mu }^{2}$ with the actual variance $\sigma_{\mu }^{2}$ (following the approach in Bonnen et al. (2015) where the actual variance of the stimulus noise is used in data analysis). In our study, a period of adaptation to the continuous stimulus environment was allowed so that, among other reasons, the participant’s estimates of noise in the world could, presumably, come close to the actual values. Analysis was held off for data collected during this period. The assumption of fixed noise during a data collection session follows (Bonnen et al., 2015). It may be justifiable by the fact that stimulus noise variance is constant during a session and that there is a period of adaptation to the stimulus before data collection begins, allowing internal noise estimates to settle.

#### The Dependent Variable, y

The part of the stimulus that is under the control of the participant—the dependent variable—is updated via the (noisy) actions of the participant as follows

(A.7)
$$y_{t}=y_{t-1}+a_{t-1}+\lambda_{t}\;\;\;\;\;\;\;\;\;\;\;\;\;\;\;\;\;\;\;\;\lambda_{t}∼N(0,\sigma_{\lambda }^{2})$$

where $a_{t-1}$ represents the (ideal) actions of the participant planned during the previous iteration and $\lambda_{t}$ represents noise in the action system. The transduction into a sensory state follows that of the independent variable.

(A.8)
$$Y_{t}=y_{t}+\psi_{t}\;\;\;\;\;\;\;\;\;\;\;\;\;\;\;\;\;\;\;\;\psi_{t}∼N(0,\sigma_{\psi }^{2})$$

Using these equations, the equivalent of equations A.4 and A.6 for the dependent variable are

(A.9)
$$p(Y_{t}|{\hat{y}}_{t})p({\hat{y}}_{t})=g({\hat{y}}_{t};Y_{t},\sigma_{\psi }^{2})g({\hat{y}}_{t};{\hat{y}}_{t-1}+a_{t-1},{\hat{\sigma }}_{\lambda }^{2})$$

and

(A.10)
$${\hat{y}}_{t}=(1-k_{y})({\hat{y}}_{t-1}+a_{t-1})+k_{y}Y_{t}\;\;\;\;\;\mathrm{where}\;\;\;k_{y}=\frac{{\hat{\sigma }}_{\lambda }^{2}}{{\hat{\sigma }}_{\lambda }^{2}+\sigma_{\psi }^{2}}.$$

just as in Figure 3.

As with the independent variable, an experimenter can use $y_{t}$ in place of $Y_{t}$ and use $\sigma_{\lambda }^{2}$ in place of ${\hat{\sigma }}_{\lambda }^{2}$ for the purposes of data analysis.

This update equation for $\hat{y}$ , assumes knowledge of the intended actions of the participant a . There is strong evidence in the literature for, at least certain, perceptual systems receiving efferent copies of action signals from action areas of the brain and incorporating those signals into their activities (Cullen, 2004; Medendorp, 2011; Straka et al., 2018). The anticipation of eye movements and a model of how those eye movements will change the retinal signal, for example, allows for the percept that the world is stationary even though retinal inputs vary constantly due to involuntary and voluntary saccades (Zimmermann & Bremmer, 2016).

Note that the update equation for $\hat{y}$ can be re-written as ${\hat{y}}_{t}={\hat{y}}_{t-1}+a_{t-1}+k_{y}\lambda_{t}$ . Comparing with the update equation for y in the world, $y_{t}=y_{t-1}+a_{t-1}+\lambda_{t}$ , it is clear that the $\hat{y}$ time series will be a “less noisy” version of the actual time series for the dependent variable as the noise is multiplied by $k_{y}$ and $k_{y}\leq 1$ . This makes sense as the estimation is based on an internal model of the dependent variable which has knowledge of how the participant intends to change the dependent variable and also takes sensory inputs into account but weights them according to how noisy the sensory system is relative to total noise. This makes the $\hat{y}$ time series trace the intended, damped-spring-like actions of the participant more closely than the raw data does.

#### Interaction Between x and y

Understanding the nature of the interaction between the independent and dependent variable is generally the aim of psychophysical experiments, for example, revealing how the direction of a moving background affects the direction that a target needs to go in to appear to be moving vertically. Here the interaction is left as unknown and is simply represented by the function f . Mapping this function will be the aim of experiments.

(A.11)
$${\tilde{y}}_{t}=f({\hat{x}}_{t},{\hat{y}}_{t})+\phi_{t}\;\;\;\;\;\;\;\;\;\;\;\;\;\;\;\;\;\;\;\;\phi_{t}∼N(0,\sigma_{\phi }^{2})$$

This perceived y is assumed to drive the actions of the participant.

#### Action

For the action component of our model, we use a damped spring model. This is based on the assumption that continuous adjustments are made to the dependent variable using the hands of the participant as they operate a mouse, joystick, or similar device. The choice of the damped spring model follows literature demonstrating the validity of such a model in the area of handwriting (André et al., 2014; Hollerbach, 1981) and other actions performed with the hands (Borst & Indugula, 2006; de Lussanet et al., 2002; Speich et al., 2005). We also found that the damped spring model produced a close fit to data coming from our particular experiment (see Figure 6). The model predicts that actions will be updated by the participant in the following way.

(A.12)
$$\Delta a_{t}=-b{\tilde{y}}_{t-\Delta t}-ca_{t-1}$$

All terms can be thought of as “forces” that have each been divided by “mass” so that we are left with only the “acceleration” components e.g. the term on the LHS represents an acceleration of the dependent variable as a is a “velocity” i.e. it represents a change in the dependent variable. The LHS term can be considered the (acceleration component of the) force driving the intended action of the participant. This force is composed of two parts. The first RHS term represents the “restoring force” and the second term represents the “damping force” of our spring. Thus, b is a spring constant and c is a damping constant. Note that Δt is used, in theory, to represent the time taken for perceptions to turn into actions, but as it is the only time lag in our set of equations so far it is, in reality, a catch-all time buffer between stimulus changes for the independent variable and the initiation of responses to those changes.

It is important to note that using $\tilde{y}$ in this way means that the aim of the participant is to make $\tilde{y}$ equal to zero as the first RHS term, representing the spring’s “restoring force,” is a negative linear function of how far $\tilde{y}$ is from zero. This constrains the function f in equation A.11. The output must reflect how far perceived y is from the goal of the participant during the experiment.

Rearranging leads to the following update equation for the action system:

(A.13)
$$a_{t}=(1-c)a_{t-1}-b{\tilde{y}}_{t-\Delta t}.$$

## Appendix B

### Analysing Experimental Data

#### New x

According to our model, it is an estimated version of x (denoted $\hat{x}$ ) that is fed into the xy interaction mechanism. This corresponds more closely to an independent variable in a trial-based experiment as errors due to continuously changing the stimulus are accounted for as well as possible. Setting ${\hat{x}}_{0}=x_{0}$ and using the sensory noise estimate, $\sigma_{\omega }^{2}$ , from the training session and the variance of the random walk, $\sigma_{\mu }^{2}$ , to calculate the weight given to sensory data $k_{x}$ , it is possible to iterate the $\hat{x}$ update equation, ${\hat{x}}_{t}=(1-k_{x}){\hat{x}}_{t-1}+k_{x}x_{t}$ , to get the $\hat{x}$ time series which we propose is equivalent to x ’s from a trial-based experiment. This trial-based equivalent version of x is marked with an asterisk.

New x:

(B.1)
$$x_{t}^{*}={\hat{x}}_{t}$$

#### New y

Similarly, applying the $\hat{y}$ update equation ${\hat{y}}_{t}=(1-k_{y})({\hat{y}}_{t-1}+a_{t-1})+k_{y}y_{t}$ (equation A.10) will produce y values that more closely resemble dependent variable values from a trial-based experiment. This equation requires knowledge of the intended actions, a , and the action noise, λ i.e. a sense of what parts of the adjustments made by the participant, captured in y , might be considered intentional and what parts are noise. The updated equation for the independent variable, $y_{t}=y_{t-1}+a_{t-1}+\lambda_{t}$ , says that the change in the dependent variable is a sum of the intended action and additive Gaussian noise. Therefore, the intended actions of the participant ( a ) are the dependent value changes stripped of additive Gaussian noise. Accordingly, they should be revealed by a process that finds the average response of the participant to a stimulus change. To uncover the general properties of the intended action system we can turn, then, to the average response of the participant to a step in the training session. As it turns out in our experiment, this was well modeled using our damped-spring equation. This equation, with the spring ( b ) and damping ( c ) constants found in the training session, can be applied to the participant response series y to parse it into intended and unintended (the remaining) parts.

To apply this model, described in equation A.13, one needs to know ${\tilde{y}}_{t}$ , the perceived distance of y from its goal value, for all t . Here we can apply a very simple principle, that is, that an intention (here, the closing of the current gap between y and goal y ) takes time to be realized due to lags in the action system. We’ll denote this time lag $\Delta t^{\prime }$ . Note that this is different from Δt in equation A.13 which represents the time between a theoretically noticeable independent variable deviation from a goal value and the initiation of spring-like action (i.e. Δt precedes $\Delta t^{\prime }$ ). By this principle, the current y setting is a result of a discrepancy between y and goal y initially acted upon $\Delta t^{\prime }$ seconds ago. This would make the $\tilde{y}$ driving current behavior (that is ${\tilde{y}}_{t-\Delta t}$ in our model) equal to the gap between current y and y occurring $\Delta t^{\prime }$ seconds in the future, i.e. ${\tilde{y}}_{t-\Delta t}=y_{t}-y_{t+\Delta t^{\prime }}$ . This can be applied to equation A.13 and the equation iterated to produce a time series for a —the intended actions of the participant (note, that the first Δt seconds of data will be lost in the process).

As all other variables are known it is possible to use an optimization method to find the $\Delta t^{\prime }$ that produces the best fit. Note that this principle misses possible action changes due to changes in $\tilde{y}$ during the time lag $\Delta t^{\prime }$ but this might be accounted for with a more sophisticated action model. The simple version is correct on average, as the driver of changes in $\tilde{y}$ are changes in the independent variable x and these changes are random with a mean of 0, but it potentially misses some details.

An action noise time series can then be produced using $y_{t}=y_{t-1}+a_{t-1}+\lambda_{t}$ (equation A.7). The variance of this noise and the sensory noise from the training session can be used to calculate $k_{y}$ and equation A.10 can finally be used to produce $\hat{y}$ which are the y values fed into the xy interaction mechanism.

The interaction between x and y is represented by f in equation A.11. Recall that it produces $\tilde{y}$ representing the sensed deviation of y from some goal value. This deviation is due to the use of a continuous psychophysics regime; if the stimulus didn’t change so quickly and the participant was given more time to respond, these discrepancies between the current y and where the participant thinks y should be shouldn’t occur. So a final step in the analysis is to remove those discrepancies. Removing them is simple if the perceived dependent variable y is a linear function of the estimated dependent variable $\hat{y}$ . In our experiment shifting the actual target direction by, say, 10∘ clockwise produces a perceived shift of approximately the same magnitude so a y with perceived errors removed can be calculated as follows:

New y :

(B.2)
$$y_{t}^{*}={\hat{y}}_{t}-{\tilde{y}}_{t}$$

An experimenter would need to know the nature of the $\hat{y}\tilde{y}$ relationship and use an appropriate equation here. Note that this step eliminates a further component of the time lag between the dependent and independent variable time series as ${\tilde{y}}_{t}$ incorporates changes due to the time lag Δt . But it is possible to also perform normalized cross-correlation on the two series to find any remaining time lag denoted $\Delta t^{*}$ . This step, if taken, is based on the assumption that dependent and independent variables should be correlated.

$x^{*}$ and $y^{*}$ may be considered equivalent to x and y in a trial-based experiment and may, thus, be used to assess the equivalence of results from the two methods.

## Appendix C

### Bayesian Approach to the Action System

The action-driving system embodied by equation A.13 where future actions are determined based on current perceptions, actually takes into account a model of the participant’s action system. It does *not* make changes to a so that next-iteration actions immediately negate the current discrepancy between the perceived dependent and goal-dependent values. Instead, it makes changes to a with the physical constraints of the action system “in mind,” that is, it acknowledges the damped spring-like properties of the action system and adjusts a accordingly. In this sense it is attempting to maximize the probability of zero discrepancy between the perceived and target dependent value given that the action system will behave in a certain way. This includes the assumption that there will be a mean change of zero in the independent component of the stimulus during the time that the action is performed (based on equation A.1). In this sense, the actions are optimal within the probabilistic and physical constraints of the system.

Evidence for the damped-spring action model was shown to come from the training session data in our experiment (see Figure 6). It can also be shown to arise from casting the decision process in terms of BDT—a theory describing real or ideal systems that make Bayes-optimal decisions under noisy conditions (Parmigiani et al., 2001). BDT assumes that actions are chosen to maximize expected utility denoted here by E(utility) . The optimal action $a^{*}$ at any given iteration is calculated as follows (Körding & Wolpert, 2006):

(C.1)
$$a^{*}=\mathrm{argmax}E(\mathrm{utility})$$

where

(C.2)
$$E(\mathrm{utility})=\sum_{\mathrm{possibleoutcomes}}u(\mathrm{outcome})p(\mathrm{outcome}|a)$$

where u(o) is a utility function describing the worth of an outcome, o . This function takes the energetic costs and the reward value of outcomes into account (Körding & Wolpert, 2006).

The outcome of interest for a participant is the perceived dependent value $\tilde{y}$ as this is the entity for which a goal value has been assigned. This value drives the actions of the participant.

Although it is impossible to know the utility of particular outcomes $\tilde{y}$ to the participant, the data provides hints. Assuming the participant has an effective action system, the utility of an outcome ought to be reflected in the distribution of those outcomes over a data collection session; more desirable outcomes should occur more often and less desirable outcomes less often.

The $\tilde{y}$ distribution that is most closely tied to the desires of the participant is the distribution of $\tilde{y}$ values calculated as in Appendix B. Each $\tilde{y}$ value in this distribution reflects how the participant changed the dependent value in the time it typically takes the action system to change this value to the goal value.

This $\tilde{y}$ distribution is plotted on the left in Figure 12 for the data collection session graphed in Figure 8. On the same graph is drawn the best-fitting Gaussian. The plot indicates that participant ED places highest value on $\tilde{y}$ values close to zero (mean = -0.07∘, 95% CI: -0.66∘ to 0.52∘) and that value falls off following a roughly Gaussian distribution. This distribution can be thought of as representing the utility of particular $\tilde{y}$ values to the participant filtered through a noisy action system.

The expected utility is maximized when the profiles of the two distributions on the right-hand side of equation C.2 closely match one another. The $\tilde{y}$ distribution just described is an estimate of u(outcome) . Is the second distribution, p(outcome|a) comparable?

Over an entire data collection session the latter distribution equates to the probability of $\tilde{y}$ values given a certain action system or set of policies governing the actions of the participant. For a damped spring policy or action system, the $\tilde{y}$ distribution of interest is the distribution of the $\tilde{y}$ values taken as driving the damped spring. These are the $\tilde{y}$ values “causing” the “intended” actions a under our BP model. These are plotted in Figure 12 for the same session as that for the u distribution in Figure 12. This distribution, too, is centered on zero (mean = 0.02∘, 95% CI: -0.67∘–0.72∘) and is roughly Gaussian with a similar spread. The close match between these two distributions indicates that the damped spring action policy comes close to maximizing expected utility.

## Appendix D

### Continuous Tracking Version of the PB Model

It is a simple matter to adjust the model and procedure to deal with continuous-tracking experiments like that used in most continuous psychophysics studies published to date (Bonnen et al., 2015, 2017; Burge & Cormack, 2020; Lakshminarasimhan et al., 2020; Grillini et al., 2021; Straub & Rothkopf, 2022) where the task of the participant is not to adjust the stimulus to meet a certain goal but, simply, to continuously report the state of the stimulus. In a two-variable version of a continuous-tracking experiment the actions of the participant do not affect the dependent variable y , but, instead, affect a variable that represents the perception of the dependent variable which we can label ${\tilde{y}}^{\prime }$ . In Bonnen’s study, for example, this was the position of a cursor (that the participant was supposed to keep on the center of a Gaussian blob). In the case of our experiment that might look like asking the participant to adjust the position of a Gaussian blob on the outside of the display area to match the direction the target appears to be going in, rather than asking them to adjust the target so that it always appears to go in a specific direction. In that case, equations A.7 and A.10 would need to be adjusted to reflect the fact that the actual dependent variable value does not change (although, like the independent variable, noise could be added to it making equations equations A.7 and A.10 exactly equivalent to the equations for x ). The dependent data is no longer composed of both “intended” actions and noise which simplifies the mathematics although it remains useful to identify the “intended” component of the participant’s noise-laden target tracking actions as this more closely reflects their perceptions of the dependent variable. To calculate this, in equation A.11, $\tilde{y}$ would be replaced with ${\tilde{y}}^{\prime }$ and in equation A.13, ${\tilde{y}}_{t-\Delta t}$ would be replaced with some variable that reflects how far current ${\tilde{y}}^{\prime }$ is from ${\tilde{y}}^{\prime }$ at $\Delta t^{\prime }$ seconds in the future.

## Appendix E

### Stimulus Details

The target consisted of 30 Gabor patches evenly distributed around a 4∘ radius ring centered on the display. The stimulus was displayed on a SONY Trinitron G420 monitor ( 1024×768 pixels at 100 Hz) that was placed 60 cm from a chin rest. Observers were instructed to fixate as close to the center of the ring as possible (a fixation point was not included as it would provide an unwanted reference for judging target motion). The background consisted of 40 Gabor elements randomly scattered over a 20∘ radius region centered on the display (elements were free to also appear inside the target ring). All Gabor elements were distributed and oriented randomly at the beginning of each continuous session. The Gaussian envelopes had a standard deviation of 8‘, and the carrier had a spatial frequency of 3 cycles per degree. All Gabor elements had a Michelson contrast of 0.40 ([Lmax-Lmin]/[Lmax+Lmin]). Each Gabor element’s phase drifted at a rate that depended on its orientation and the speed and direction of the target or background object to which it belonged. For example, if the Gabor element was part of the background, its rate of drift was the dot product of a unit vector representing the direction of drift (orthogonal to the Gabor’s stripes) and a vector representing the speed and direction of the background. This is equivalent to making all background Gabor elements drift consistently with the “intersection of constraints” (Adelson & Movshon, 1982) solution for the desired background motion. The target was separately constructed in the same manner. This produced the percept of two rigidly moving objects, one being the target and the other the background. Both “moved” in this way at a speed of 6∘/s.

For the background, a random walk (50% chance of a clockwise or anticlockwise 2∘ step every 100 ms) was selected that allowed the background to cover a desired direction span; 0∘ (rightwards) to 90∘ (upwards) for the first experiment and 0∘ to 180∘ for the second “symmetric” experiment (+ 10∘ buffer either side of the extremes). During the session (2 minutes 10 seconds for the first and 4 minutes 10 seconds for the second experiment) the task of the participant was to adjust the target direction so that it appeared to move vertically upwards as well as possible at all times. Adjustments were made using a mouse where leftward movement adjusted the direction anticlockwise and the rightward clockwise.

Stimuli were generated and updated using Unity version 2018.4.7f1 running on a PC with a Windows 10 operating system.

Participants consisted of three experienced observers recruited from the Vision Lab at the University of Western Australia. All participants except MF were naïve to the hypothesis of the experiment. All participants had normal or corrected-to-normal visual acuity. Observer ED has a divergent squint and completed the experiments using an opaque eye patch over the nondominant eye. All participants gave written informed consent prior to beginning the experiments. These experiments had ethics approval (RA/4/1/4503) from the Human Ethics Committee at the University of Western Australia.
